# Supplementary material for: Emotional skills and health assessment in interventions for intimate partner violence perpetrators: A systematic review of randomized controlled trials
Source: PLoS One. 2025 Jul 9;20(7):e0328034. doi: 10.1371/journal.pone.0328034 (PMC12240372; doi:10.1371/journal.pone.0328034)
Supplement: S2 File — (ZIP) [file pone.0328034.s002.zip › Supporting information/Appendix_Summary of Trials.docx]

Appendix.

Main characteristic compilation of the final sample.

|  | Sample size (N)  Recruited population Mean age year (M)  Court referred or Volunteers  (% of men) | Intervention setting/staff | Emotional intervention  Intervention group (IG) | | | Standard or other intervention  Control group (CG) | | Follow-up period | Outcomes  (Main tools used) | Results |
| --- | --- | --- | --- | --- | --- | --- | --- | --- | --- | --- |
|  |  |  | Treatment  type and intervention format (n) | Type of emotional skills delivered | N Sessions Intervention length | Treatment type and intervention format (n) | N Sessions Intervention length |  |  |  |
| Cavanaugh et al., (2011)  USA | N = 55 attendees for a local anger management program (potentially at risk of IPV)  M = 57.1% were  between the ages of 18 and 35, and 42.9% were over age 36  Volunteers  (100% men) | Community centers/  Master-level mental health professional | Dialectical Psycho-educational Workshop (DPEW) Group  (n = 28) | Emotional regulation and Empathy | 1 session (2 hours) | Anger management program. Group  (n = 27) | 1 session (2 hours) | 1 month (Evaluation feedback) | 1. Anger management skill (STAXI) 2. Empathy (BEES) 3. Self-controlling (WCQ) 4. Risk of eruptive violence (REV) | The IG increases awareness of adaptive coping skills, anger management, empathy, and decreases potential risk for expressions of physical violence |
| Cotti et al., (2020)  USA | N = 154 convicted of intimate partner violence (misdemeanor offenders)  M = 32.5  Court referred  (60.4% men) | Community centers/  State-certified therapists | Cognitive Behavioral Therapy (CBT)  Group  (n = 77) | Emotions management (stress, anxiety and anger) | 16 sessions  9 months | Duluth model  Group  (n = 77) | 21 sessions  9 months | 3 years | 1. Official recidivism (OR) | IPV recidivism rate was 14 pp higher for offenders (men) assigned to CG |
| Dunford et al., (2000)  USA | N = 644 married U.S. Navy couples  (misdemeanor offenders)  M = 27  Court referred  (Mostly men) | Military/  FAC-Navy staff and University of Colorado research team | Cognitive Behavioral Therapy (CBT)  Group  (n1 = 168)  (n2 = 153)  Individual  (n3 = 173) | Emotions management (anger and jealousy) and Empathy | IG1-2: 32 sessions (26 weekly + 6 monthly) IG3: 12 monthly sessions 12 months | Individual security planning  (n =150) | 0 sessions 12 months | 6 months | 1. Official recidivism (OR)  2. Couples recidivism (CR) 3. Abusive behaviors  (MCTS) | No significant differences between groups |
| Easton et al., (2018)  USA | N = 63 arrested for IPV with substance dependence (SUD)  M = 39.4  Court referred  (100% men) | Community centers/  Master-level therapists | Substance Abuse-Domestic Violence therapy (SADV). Individual  (n = 29) | Emotions management (anger and negative mood states) | 12 weekly sessions  3 months | Drug counseling  Individual  (n = 34) | 12 weekly sessions  3 months | 3 months | 1. State-Trait Anger Expression (STAXI) 2. Working Alliance (WAI) 3. Abusive behaviors (CTS-2) 4. Aggressive behaviors and hours of partner contact each day (TLFBSV) | SADV participants (IG) were less likely to engage in aggressive behavior proximal to a drinking episode, and reported fewer episodes of violence than CG participants at posttreatment follow-up. |
| Fernández-Montalvo et al., (2019)  Spain | N = 70 patients in a drug addiction program (SUD) with IPV episodes  M = 35.7  Volunteers  (58,6% men) | Community centers/  Clinical psychologists with 10 or more years of experience in addictions | Integrated intervention program for drug addiction and IPV. Group  (n = 34) | Emotions management (anger and emotional expression) and Empathy | 36 sessions (with 20 additional IPV sessions 90-min)  12 months | Usual treatment in a drug addiction program (TAU).  Group  (n = 36) | 36 sessions (weekly-biweekly 45-60 min)  12 months | 6 months | 1. Couples recidivism (CR) 2. Abusive behaviors (CTS-2) 3. Distorted thoughts about the use of violence (IDT-V) 4. Anger management skill (STAXI) 5. Impulsivity (BIS-10) 6. Maladjust-ment Scale | The IG showed an IPV success rate significantly higher than CG and both groups achieved improvements in associated variables |
| Gilchrist et al., (2021)  UK | N = 104 offenders in substance use treatment  M = 42.1  Volunteers  (100% men) | Community centers (substance use service)/ Health-care professionals | ADVANCE intervention (IPV+TAU)  Individual/ Group  (n = 54) | Emotional regulation and Empathy (perspective taking) | 16 sessions (2-4 individual sessions + 12 group sessions)  16 weeks | Usual substance use treatment (TAU)  Individual/ Group  (n = 50) | 16 sessions (Typically, fortnightly individual sessions + weekly group sessions) 16 weeks | Post-Intervention | 1. Couples recidivism (CR) 2. Intimate partner abuse (URICA-DV...) 3. Substances use (AUDIT…) 4. Mental health  (PHQ-9, GAD-7…) 5. Self-control (BSC) | Neither substance use nor IPV perpetration had worsened for IG.  Both groups improve in mental health, although the IG scores 10 pp less than the CG among those who scored higher on the scales |
| Hesser et al., (2017)  Sweden | N = 65 participants who report significant difficulties with aggression (mild form) in a close stable relationship  M = 36.9  Volunteers  (43% men) | Internet/  Clinical psychology students with clinical training | Internet-delivered cognitive behaviour therapy (iCBT)  Individual  (n = 32) | Emotional regulation (homework exercises) | Guided self-help format (therapists provided 24h feedback)  8 weeks | Monitored waitlist control  Individual  (n = 33) | 8 weeks | 1 year | 1. Violent behaviour (CTS-2 and MMEA) 2. State/trail aggression (AQ) 3.Relationship quality (DAS) 4. Emotion regulation strategies (DERS)  5. Anger rumination (ARS)  6. Anxiety and depression symptoms (GAD-7 and PHQ-9) | The IG had significantly reduction in emotional abuse, anxiety and depression symptoms.  The treatment effect was partially mediated by changes in emotion-regulation ability |
| Kraanen et al., (2013)  Netherlands | N = 52 adults in SUD treatment with physical IPV  M = 36.18  Volunteers  (69% men) | Community centers/Social workers with experience in substance abuse counseling | I-Stop (substance abuse and IPV)  Individual  (n = 27) | Emotional regulation (anger management and coping with emotions that may lead to substance use and IPV). | 16 sessions  16 weeks | CBT-SUD+ (only 1 IPV session) Individual  (n = 25) | 16 sessions  16 weeks | Post-intervention | 1. Abusive behaviors (CTS-2) 2. Substance use (TLFP…) 3. Psychopa-thology (BSI) 4. Marital Satisfaction (MMQ) | No significant differences between groups (Both groups improve in substance use and IPV perpetration) |
| Labriola et al., (2005)  USA | N = 420 offenders arraigned on a domestic violence misdemeanor  M = 31  Court referred  (100% men) | Community centers/ Community centers staff | Duluth model (batterer program + monthly judicial  monitoring; batterer program + graduated monitoring Group  (n1 = 102;  n2 = 100) | Emotional regulation  (recognizing emotions and controlling anger) | 26 weeks | Judicial monitoring (monthly and graduated) Individual  (n1 = 109;  n2 = 109) | 26 weeks | 1 year | 1. Official recidivism (OR)  2. Couples recidivism (CR) 3. Abusive behaviors  (CTS) | No significant difference between groups |
| Nesset et al., (2020)  Norway | N = 125 offenders who voluntarily seeking treatment  M = 60.5  Volunteers  (100% men) | Community centers (outpatient health service)/ Psychiatric nurses (for IG) and specialists in clinical psychology and education (for CG) | Cognitive Behavioral Therapy (CBT)  Individual + group  (n = 67) | Emotional regulation  (dysfunctional anger and negative emotions) | 2 individual sessions +  15 group sessions  15 weeks | Mindfulness-based stress reduction  Individual + group  (n = 58) | 1 individual session +  8 group sessions  8 weeks | 1 year | 1. Couples recidivism (CR)  2. Abusive behavior (CTS-2)  3. Symptoms of emotional distress (HSCL-25) 4. General emotion regulation (DERS) | No significant difference between groups.  A substantial risk estimate reduction was found in both groups.  Both groups had a reduction of anxiety and depression (symptom scores remained high) and in difficulties in emotion regulation |
| Romero-Martínez et al., (2022)  Spain | N =51 convicted of intimate partner violence  M = 44.4  Court referred  (100% men) | Community centers/ Therapists with one or more years of experience with batterer interventions | Standard Intervention Program (SIP) + Cognitive training  Group  (n = 20) | Empathic and emotion-decoding abilities | 35 (2h) + 31 (15 min) sessions (2xWeek)  9 months | SIP + Placebo training  Group  (n = 31) | 35 (2h) + 31 (15 min) sessions (2xWeek)  9 months | Post-intervention | 1. Processing speed (CPT-III) 2. Emotion decoding (Eyes test) 3. Risk of recidivism (SARA) | Only IG improved their processing speed and cognitive flexibility.  The IG presented the lowest risk of recidivism. |
| Rosenfeld et al., (2019)  USA | N = 109 stalking offenders  M = 36.05  Court referred  (96% men) | Community centers/ Graduate students in clinical psychology trained to study | Dialectical Behavior Therapy (DBT)  Individual + group  (n = 57) | Emotional regulation (manage of strong emotions and urges) | 48 sessions  (24 group + 24 individual)  24 weeks | Cognitive Behavioral Therapy (CBT) Individual  (n = 52) | 18 sessions  18 weeks | 6 months | 1. Official recidivism (OR)  2. Aggression (AQ)  3. Impulsivity (BIS-11) 4. Anger (STAXI) 5. Empathy (EQ) | No significant differences between groups on re-offense (recidivism). Small and significant effect in Empathy |
| Stover et al., (2019)  USA | N = 62 fathers with a history of IPV who are in substance use disorder (SUD) treatment  M = 35.85  Most of them were court referred  (100% men) | Community centers/ Master´s level clinicians trained to study | Fathers for Change (F4C). Integrated intervention IPV + child maltreat-  ment + TAU Individual  (n = 33) | Emotional regulation | 16 sessions (12 weekly sessions + 4 booster sessions)  12 weeks | Dads and Kids (DNK). Psycho-educational intervention (behavioral parenting skills). Individual  (n = 29) | 16 sessions (12 weekly sessions + 4 booster sessions)  12 weeks | Post-intervention (3 months) | 1. Abusive behaviors (CTS-2  2. Difficulties in emotion regulation (DERS) 3. Anger management skill (STAXI) | No significant differences between groups on IPV. IG showed some benefit over CG in affect dysregulation symptoms and substance use relapse. |
| Zarling et al., (2015)  USA | N = 101 adults in mental health treatment with IPV episodes  M = 31  Volunteers  (32% men) | Community centers (mental health services)/ Psychology doctoral students | Acceptance and Commitment Therapy (ACT). Group  (n = 50) | Emotional Intelligence | 12 sessions  12 weeks | Support and discussion format. Group  (n =51) | 12 sessions  12 weeks | 6 months | 1. Emotional abuse (MMEA) 2. Physical aggression (CTS-2) 3.Experiential avoidance (AAQ) 4. Difficulties in emotion regulation (DERS) | The IG had significantly greater declines in psychological and physical aggression, and reductions in experiential avoidance and emotion dysregulation |
| Zarling et al., (2022)  USA | 338 convicted of IPV.  M = 33.83  Court referred  (100% men) | Community centers/ Qualified facilitators trained in ACT or Duluth Model approach | Acceptance and Commitment Therapy (Third-wave CBP to increase psychological flexibility)  Group  (n = 171) | Emotional regulation and Empathy (perspective-taking) | 24 sessions  24 weeks | Duluth model  Group  (n = 167) | 24 sessions  24 weeks | 1 year | 1. Official recidivism (OR)  2. Couples recidivism (CR)  3. Physical aggression (CTS-2)  4. Controlling behaviors (CBS)  5. Stalking behaviors (SBC) | No difference between groups in IPV charges (but victim reports indicated that IG engaged in fewer IPV behaviors). IG participants incurred fewer other charges. |

*Note*. ARS = Anger Rumination Scale; AQ = Aggression Questionnaire; AAQ = Avoidance and Action Questionnaire; AUDIT = Alcohol Use Disorders Identification Test; BEES = Balanced Emotional Empathy Scale; BIS-10 = Barratt Impulsiveness Scale; BSC = Brief Self Control Scale; BSI = Brief Symptom Inventory; CBS = Controlling Behaviors Scale; CG = control group; CPT-III = Continuous Performance Test-III; CTS-2= Revised Conflicts Tactics Scale; DAS = Dyadic Adjustment Scale; DERS = Dysfunctional and Emotional Regulation Scale; EG = Empathy Questionnaire; GAD-7 = General Anxiety Disorder-7 ; HSCL-25 = Hopkins Symptom Checklist 25; IG = intervention group; IDT-V = Inventory of Distorted Thoughts About the Use of Violence; IPV = intimate partner violence; MCTS = Modified Conflicts Tactics Scale; MMEA = Multidimensional Measure of Emotional Abuse; MMQ = Maudsley Marital Questionnaire; PHQ-9 = Patient Health Questionnaire-9; REV = Risk or Eruptive Violence Scale; SARA = Spouse Assault Risk Assessment; SBC = Stalking Behavior Checklist; STAXI = State-Trait Anger Expression Inventory; SUD = substance use disorder; TAU = addiction treatment as usual; TLFB = Timeline Follow-Back Interview; TLFBSV = Timeline Follow-Back Spousal Violence; URICA-DV = Rhode Island Change Assessment for Domestic Violence Offenders-Revised; WAI = Working Alliance Inventory; WCQ = Ways of Coping Questionnaire.
