# Supplementary material for: Emotional skills and health assessment in interventions for intimate partner violence perpetrators: A systematic review of randomized controlled trials
Source: PLoS One. 2025 Jul 9;20(7):e0328034. doi: 10.1371/journal.pone.0328034 (PMC12240372; doi:10.1371/journal.pone.0328034)
Supplement: Attachment — Submitted filename: PONE_review_10.20.24.pdf [file pone.0328034.s004.pdf]

This paper provides a systematic review of randomized controlled trials (RCTs) examining interventions for intimate partner violence (IPV) offenders. I have one primary concern and a suggestion for improving the study.

My main concern with this systematic review is that certain aspects of the research question are ambiguous, such as the study sample. Some studies included both male and female participants, others focused on individuals with diagnosed substance use disorders, some included court-referred participants, and one study involved participants from a military setting. Comparing intervention outcomes across such diverse populations is problematic, as it risks conflating fundamentally different participant groups—essentially comparing apples to oranges. The authors should provide more detail on how they account for these sample differences in review and in drawing their conclusions.

Moreover, to enhance the clarity of the review, it would be beneficial for the authors to include a summary table that outlines the key elements of each study (e.g., type of intervention program, main outcomes assessed, program duration, follow-up period, etc.), along with their findings and conclusions. Although these details are discussed in the text, a well-organized table would help structure the information and make it easier for readers to follow. Without such a table, it is challenging to track the findings of individual studies in relation to their other characteristics.
