# Supplementary material for: Emotional skills and health assessment in interventions for intimate partner violence perpetrators: A systematic review of randomized controlled trials
Source: PLoS One. 2025 Jul 9;20(7):e0328034. doi: 10.1371/journal.pone.0328034 (PMC12240372; doi:10.1371/journal.pone.0328034)
Supplement: Attachment — Submitted filename: Response to Reviewers.pdf [file pone.0328034.s006.pdf]

Emotional Skills and Health Assessment in Interventions for Intimate Partner Violence Perpetrators: A Systematic Review of Randomized Controlled Trials

Manuscript ID PONE-D-24-21114

Dear Dr. Ventura,

Thank you for granting us the opportunity to further revise our manuscript titled “Emotional Skills and Health Assessment in Interventions for Intimate Partner Violence Perpetrators: A Systematic Review of Randomized Controlled Trials", and resubmit it to PLOS ONE.

We have carefully addressed the issues raised by the reviewers, and a detailed point-by-point response is provided on the following pages. Revisions to the manuscript have been marked in the upload file 'Revised Manuscript with Track Changes'. An unmarked version of our revised paper without tracked changes has been also uploaded in a separate file labeled 'Manuscript'.

If you have any additional questions or further requests, please do not hesitate to contact us. We look forward to your feedback.

Sincerely,

The authors

Journal requirements’

Comment 1. Manuscript revision according PLOS ONE's style requirements.

Response: The manuscript has been revised to fully comply with PLOS ONE's style guidelines.

Comment 2. Data Availability Statement.

Response: We have carefully reviewed the requirements for compliance with the statement, "All relevant data are within the manuscript and its Supporting Information files." The procedure has been detailed in the manuscript, including methodological aspects that facilitate replication of the study. Additionally, a dataset has been included in the submission to complete the "Data Availability Statement," ensuring the replicability of all findings reported in the article (see comment and response 3). These materials have been uploaded as Supporting Information files.

Comment 3. Policy on Data Availability.

Response: A table containing the data extracted from the primary research sources for the systematic review has been included as supplementary information. Additionally, an Excel file has been provided, detailing all studies identified during the literature search, including those excluded from the analyses. For each excluded study, the reason(s) for exclusion are listed. All data extracted from each study included in the systematic review are provided in a supplementary file titled “Appendix_Summary of Trials.” The table presenting the completed risk of bias and quality/certainty assessments for each study or outcome is already present in the main document.

Reviewers' comments to the authors and responses

Reviewer 1

Comment 1. *Is GBV used to mean intimate partner violence or any form of gender-based violence (including non-partner sexual violence)?*

> **Response:** We sincerely appreciate this comment. The question posed by the reviewer has prompted us to reflect on one of the main constructs of our study. Indeed, the use of the term GBV throughout the document, particularly in the introduction, may lead to confusion. To enhance clarity and ensure coherence with the study's objectives and results, we have replaced both the term GBV and the explanations previously provided in association with the term intimate partner violence (IPV). IPV refers to abuse or aggression that occurs in a romantic relationship and encompasses both current and former spouses and dating partners. It includes physical, verbal/symbolic, or sexual acts that cause—or have a reasonable potential to cause—harm to an intimate partner. According to the literature, women experience this form of violence to a greater extent than men, and with more severe consequences (WHO, 2021); however, men can also be victims (Hines, 2015; Perryman & Appleton, 2016; Scott-Storey, 2023). Therefore, a considerable portion of the reviewed studies and the arguments presented in our review focus on male perpetrators, albeit not exclusively.

Comment 2 (Introduction). *"Which are among the main proposals to prevent new acts of violence against women"—"Proposals" does not quite seem to fit here—perhaps interventions, methods, options, or another synonym? (Proposals is also used on pg. 19.).*

> **Response:** The term "proposals" has been replaced with "interventions" in accordance with the reviewer's suggestion.

Comment 3 (Results). *On page 13, programs ranging from 16- 56 sessions (weeks?) were considered long-term but a program with 12 sessions was considered short. Should those in a certain range (e.g., 20 or 23 weeks/ sessions and under) be considered "moderate" in length? (Length also mentioned on pg. 22.).*

> **Response:** We appreciate your comments and agree with your suggestion. We have adjusted our classification of the interventions and regrouped those that reported at least 24 sessions (equivalent to 6 months in duration) as long-term interventions, while those with 16 sessions have been included in the short-term intervention

category. We have also taken note of your observation regarding the use of the term "moderate," which is not commonly employed to assess the duration of interventions.

Comment 4 (Results). *Are most programs delivered once weekly for one hour? Do you have data regarding the dosage in terms of hours of treatment and number of sessions for the programs?*

Response: The requested information is presented in the table provided as supplementary material following the review.

Comment 5 (Results). *The statement "one internet-based treatment (1 week per module during 8 weeks) involving therapists who provided 24-hour feedback in a secure encrypted web page asynchronously" feels like it could use more explanation. Was the program delivered live or self-paced and then the therapists provided feedback to the participants based on their submitted work? How does feedback work in IPV intervention programs?*

Response: Thank you for your insightful comment. We believe the concerns raised by the reviewer will be addressed in the following sections of the manuscript. Specifically, when we state that "Training for a variety emotional skills was provided in the RCTs." (page 11), We refer to the findings of Hesser et al. (p. 12), which indicate that the study "…delivered cognitive behavioral therapy via the internet in the form of guided self-help involving a support therapist; the intervention incorporated emotion regulation and communication, anger rumination and conflict-resolution techniques"… Furthermore, this information is elaborated upon on page 14, where we discuss the duration of the intervention: "Regarding the duration of the intervention…one internet-based treatment (8 weeks) involving therapists who provided 24-hour feedback (via the contact handling system and determining whether the participant was ready to proceed to the next module)".

Comment 6 (Results). *Regarding control groups, many of these groups were not recognized treatments for IPV. As such, one would not expect to see a change in IPV recidivism/ behaviour as the IPV is not being addressed. ("Concerning control groups, four interventions . . . focused predominantly on the usual SUD treatment . . . psychoeducational, focused on IPV . . . behavioral parenting skills plus SUD . . . anger management program . . . CBT anger management . . . mindfulness-based stress reduction therapy."* *This seems that it would have different implications if the test groups included IPV tx + learning emotional skills versus control groups of IPV tx only. In this case, the test groups include IPV tx with emotional learning skills but the control groups do not appear to be IPV tx only.*

Response: We appreciate the point raised by the reviewer. This topic has been the subject of considerable reflection among the authors of this manuscript. After reviewing the programs evaluated in the selected articles, as well as drawing from our professional experience, we are concerned about the real challenges in assessing the success of interventions addressing this type of violence. In fact, in the manuscript (in the discussion section), we highlight that one of the issues we encountered in this systematic review, which focuses on IPV interventions that included emotional variables, is the significant variability within the control groups. For this reason, we encourage caution when interpreting the results (see page 21). Moreover, the scenario you propose—where both the intervention and control groups include IPV sessions and differ only in the development of emotional variables in the intervention group—represents the ideal situation we aim to develop in the near future.

Comment 7 (Results). *On page 18, it would be more clear to say, "Only one of the five trials that evaluated ORs found . . ."*

Response: Thank you for your comment. We have revised the wording using the suggested phrasing.

Comment 8 (Dicussion/limitations). *"the inclusion of mixed samples of men and women in some trials"—it is not stated in the descriptive data how many studies also included women and the % of men in these samples. It should be stated above, in the information about the studies, how many studies included samples of 100% men, how many were mixed, and the %.*

Response: This information is described in the "Qualitative Analysis" section (Page 10). Additionally, we have included this information in the table provided as supplementary material. Thank you for your valuable feedback.

Comment 9 (Dicussion/limitations). *"convicted men and voluntary participants" do you have data on how many studies included convicted, voluntary, or a mix?*

Response: This information is described in the "Qualitative Analysis" section (Page 10). Additionally, we have included this information in the table provided as supplementary material. Thank you for your valuable feedback.

Comment 10 (Dicussion/limitations). *Good point: "not focusing on a specific type of perpetrator of GBV" Did any of the included studies mention perpetrator typology?*

Response: We sincerely appreciate the point raised by the reviewer regarding the variability of the sample. We agree that this aspect can be seen as both a limitation of our study and one of its strengths. We have expanded on this information briefly on page 23 of the manuscript. Additionally, we would like to clarify that none of the studies mentioned specified the typology of the offenders beyond categorizing them as "misdemeanor offenders, diagnosed with substance use disorder, or those attending mental health treatment". Nonetheless, we acknowledge that it would be interesting to analyze whether IPV programs are more effective for IPV perpetrators specifically or for violent offenders in general.

Additional resources

Response: Thank you very much for pointing out these additional resources. Intervention programs that respond to the RNR model have been mentioned in the introduction (p. 4).

Reviewer 2

Major comments

Comment 1. *The choice to limit attention to RCTs is problematic. Excluding 120 out of 158 full-text articles because they used methods other than RCT feels shortsighted. RCT is often an impractical or even unethical choice of method when studying violence, as randomizing individuals who perpetrate and/or experience violence into treatment has potential to create new harms. Some studies in the sample have treatment group sizes of 28, which absolutely precludes any chance of sufficient statistical power to detect significant effects. Yet, the authors claim that such studies are more 'rigorous' than all other studies using other methods (pg 22).*

Response: We appreciate the reviewer's comment regarding the limitation to randomized controlled trials (RCTs). We recognize that this is an important consideration in the evaluation of evidence. The decision to restrict our review to RCTs is based on the intention to address some limitations reported by previous studies analyzing the effectiveness of treatments related to the control of certain biases. According to various authors (e.g., Guyatt et al., 2008), RCTs can be an optimal choice for establishing the efficacy of interventions due to their design, which minimizes biases and confounding factors. In line with the reviewer's perspective, we acknowledge the value of other study designs, such as observational

studies or cohort studies, and their ability to provide valuable information. Furthermore, we understand that including other types of studies could enrich the discussion and offer a broader perspective. In future research, we will consider the inclusion of observational studies to complement the findings of RCTs. Consequently, we have revised the manuscript to clarify the rationale for selecting RCTs, and this aspect is also mentioned as a limitation in the discussion.

Regarding the second part of your comment, we appreciate your observation about the ethical and practical considerations of conducting RCTs for the study of violence. However, the feasibility of RCTs in challenging contexts has been reviewed and supported by various studies. For instance, as highlighted in the study by Smith et al. (2020), RCTs can be designed with ethical safeguards that allow for research in sensitive contexts, provided that appropriate risk assessments are conducted. In the studies we reviewed, the majority—all except three—provided some form of assistance or intervention to the control group, thereby reducing the potential for creating new harms, or at least mitigating the attribution of such harms to the status of being in the control group. Nonetheless, we are aware of the limitation you pointed out regarding the variability of control groups, which we have acknowledged in the manuscript (in the discussion and limitations sections). We invite readers to interpret our results with caution in light of this variability. It is also important to clarify that none of the interventions reviewed, nor the research objectives, pertain to victims or populations that "experience violence."

We also thank you for highlighting the concern regarding the sample sizes of the treatment groups in some of the studies included in our review. It is important to note that several studies in the literature demonstrate that a sample size considered small does not necessarily preclude the detection of effects.

Comment 2. *The authors make the following claim as a headline finding: "Overall, the results of this systematic review indicated that more than half (53.3%) of interventions for perpetrators of GBV that included the acquisition of emotional skills were significantly more effective in reducing recidivism and improving variables associated with this type of violence than interventions that did not focus on developing these competencies." As a reader, I interpret this to mean that of those interventions that include emotional skills, 53% had larger effect sizes found than the studies of interventions that did not include emotional skills. This is a coin toss—at the sample size included in the paper, there is no way to claim a statistically significant difference between those emotional intervention studies that do and do not find larger effects. Equally half of studies on emotional interventions found larger and smaller impacts than the other studies. If my interpretation is untrue, then the paper needs to be substantially revised such that it is clear to the reader what the authors are claiming.*

If my interpretation is correct, then claiming that emotional interventions are associated with better outcomes is patently unsupported by the data.

Response: Thank you for your insights. As the reviewer pointed out, the expression of our results may appear confusing. We would like to provide the following clarifications:

1. All the trials selected for this review include emotional variables in their interventions. We have clarified in both the summary and discussion sections that 53% of the trials report evidence favoring the intervention groups when compared to control groups. Furthermore, in cases where no significant differences are found, it is often because both groups show improvement.

2. We did not state that the 53% represents greater effects, nor did we indicate that there are significant differences among the various programs as a whole, as we did not conduct a statistical analysis. The primary aim of this review is to demonstrate that, for intervention programs incorporating emotional variables, improvements are seen in the outcomes evaluated in each individual trial.

Comment 3. *In multiple places the results presented in the discussion are inconsistent with the results presented in the findings section or with such significant caveats as to undermine the initial results.*

Response: We hope that the changes made and the clarifications provided have adequately addressed this concern.

Comment 4. *Recidivism data—it is unclear how these data came about in the fifteen studies chosen. Only five studies use outcomes on recidivism from official channels, which is subject to considerable bias. The other use self-report or do not have information on recidivism. This should be addressed explicitly as anti-social behaviors are subjected to reporting bias, especially if the BIP is court mandated.*

Response: We hope that the following information clarifies the reviewer's comment: all trials provided recidivism rates or outcomes related to factors associated with intimate partner violence (IPV), including adaptive skills and the potential risk for expressions of IPV as measured by self-report scales (p. 15). Additionally, three trials obtained recidivism data through reports from partners. In total, eight trials reported recidivism data, while the remaining seven only provided information on improvements in certain variables through self-report measures.

Comment 5. *Which studies included voluntary enrollment versus court-mandated programming? How would the effects generalize to populations where BIP is required as an outcome of legal proceedings, which may directly impact participants' willingness to meaningfully engage?*

Response: The information addressing both questions can be found on page 10 of the manuscript, where it states: "Eight trials (53.3%) included court-referred participants [10,47,51,52,57,59,60,61], and the other seven trials (46.7%) included voluntary participants who were not involved in a relationship characterized by a severe form of violence or abuse; therefore, there were no convictions for IPV in those trials [50,53-56,58,62]." Additionally, relevant details are also discussed in the limitations section.

Comment 6. *The methods limit the inclusion of research articles to those studying IPV specifically, but the earlier portions of the text refer to "battering" or broader studies on violence. If IPV is the focus of this paper, specific language should be used and a definition should be provided that make clear the inclusion/exclusion criteria.*

Response: We sincerely appreciate this comment. The question posed by the reviewer prompted us to reflect on one of the main constructs of our study. Indeed, the use of the term GBV throughout the document, particularly in the introduction, may lead to confusion. To enhance clarity and ensure coherence with the study's objectives and results, we have replaced both the term GBV and the explanations previously provided with the term intimate partner violence (IPV). IPV refers to abuse or aggression that occurs in a romantic relationship and encompasses both current and former spouses and dating partners. It includes physical, verbal/symbolic, or sexual acts that cause—or have a reasonable potential to cause—harm to an intimate partner. According to the literature, women experience this form of violence to a greater extent than men, and with more severe consequences (WHO, 2021); however, men can also be victims (Hines, 2015; Perryman & Appleton, 2016; Scott-Storey, 2023). Therefore, a considerable portion of the reviewed studies and the arguments presented in our review focus on male perpetrators, albeit not exclusively.

Comment 7. *Pg 6 "e) they provided recidivism rates or described the outcomes for any factor associated with intimate partner violence (IPV)" What does this mean? It's unclear what the outcome variable inclusion criteria were.*

Response: We have made the necessary clarifications as suggested. Specifically, we have addressed point e) by providing recidivism rates and detailing outcomes

related to offenders' self-reported difficulties or deficits in variables associated with intimate partner violence (IPV), such as adaptive skills and emotional and empathic skills. This information can now be found on page 7 of the revised manuscript.

Comment 8. *Pg 10 "seven trials (46.7%) included voluntary participants who were not involved in a relationship characterized by a severe form of violence or abuse, therefore, there were no convictions for IPV in those trials" What does this mean? Why were these participants recruited?*

Response: We have clarified the information in the manuscript to specify that the participants primarily involved in the study have experienced less severe episodes of intimate partner violence (IPV) or self-reported a potential risk, as noted in only one study (Cavanaugh et al., 2011).

Minor comments

Comment 1. *Current preference is to avoid using language like "batterer" because it limits attention to physical violence. (An exception to this would be so-called 'batterer intervention programming' as this is a term of practice rather than a choice by the authors.)*

Response: Thank you for your comment. The text has been reviewed and the term changed where appropriate.

Comment 2. *"Regarding random sequence generation, two trials (13.3%) did not describe a random component in the sequence generation." Explain what this means and its significance.*

Response: Thank for your comment; this has been clarified in the manuscript (page 9).

Comment 3. *"their intervention group showed an IPV success rate" what does this mean?*

Response: Information about this issue has been added to the manuscript: the patients in the treatment group showed an IPV success rate (60.7%) that was significantly higher than that of the patients in the control group (31.6%). Regarding IPV treatment, success was defined as the complete absence of physical, sexual, and/or psychological IPV episodes.

Comment 4. *Acronyms are used without first defining them.*

Response: The acronyms have been revised.

Comment 5. *Provide more information on what the Duluth Model consists of.*

Response: Information about the Duluth Models has been added.

Comment 6. *The draft is difficult to follow in places with a number of parentheticals within the same sentence, for example.*

Response: This aspect has been reviewed in the text. Thank you for your comment.

Reviewer 3

Comment 1. *Certain aspects of the research question are ambiguous, such as the study sample. Some studies included both male and female participants, others focused on individuals with diagnosed substance use disorders, some included court-referred participants, and one study involved participants from a military setting. Comparing intervention outcomes across such diverse populations is problematic, as it risks conflating fundamentally different participant groups—essentially comparing apples to oranges. The authors should provide more detail on how they account for these sample differences in review and in drawing their conclusions.*

Response: We sincerely appreciate your insightful comments and have taken them into account to clarify various aspects of the manuscript, including the introduction, objectives, inclusion criteria, and discussion sections.

The research questions are closely related to our objectives: 1) Do intervention programs for IPV perpetrators that include "emotional skills" yield better outcomes? 2) Among the results, is the positive effect of the emotional intervention experienced by IPV offenders regarding their health and well-being evaluated? Is this positive effect proposed as a strategy to enhance motivation toward therapy? We hope that the clarification and unification of the term “IPV” in the introduction, as well as its consistent use in the objectives and other sections, will also help address these questions.

We believe that the inclusion of mixed samples is justified, as a substantial percentage of less severe IPV episodes are perpetrated by women, although it is evident that the most severe consequences of IPV are suffered by women. As we

have noted in previous responses to the reviewers, the lack of homogeneity in the sample may constitute a limitation, and the interpretation of the results should be approached with caution. However, it is also true that not focusing on a specific typology or personal characteristic of IPV perpetrators may aid in generalizing the results. Conversely, if we had concentrated solely on "apples" (e.g., convicted men), it would have significantly limited our ability to assert that the results are generalizable to any type of IPV perpetrator.

In any case, the purpose of this systematic review is to establish a solid theoretical foundation that supports the necessity or appropriateness of including emotional variables in intervention programs for IPV offenders.

Comment 2. *To enhance the clarity of the review, it would be beneficial for the authors to include a summary table that outlines the key elements of each study (e.g., type of intervention program, main outcomes assessed, program duration, follow-up period, etc.), along with their findings and conclusions. Although these details are discussed in the text, a well-organized table would help structure the information and make it easier for readers to follow. Without such a table, it is challenging to track the findings of individual studies in relation to their other characteristics.*

Response: We highly appreciate your suggestion. The table mentioned in your comment is going to be submitted as supplementary information.
