# Supplementary material for: Emotional skills and health assessment in interventions for intimate partner violence perpetrators: A systematic review of randomized controlled trials
Source: PLoS One. 2025 Jul 9;20(7):e0328034. doi: 10.1371/journal.pone.0328034 (PMC12240372; doi:10.1371/journal.pone.0328034)
Supplement: Attachment — Submitted filename: Response to Reviewers-2.pdf [file pone.0328034.s007.pdf]

Emotional Skills and Health Assessment in Interventions for Intimate Partner Violence Perpetrators: A Systematic Review of Randomized Controlled Trials

Manuscript ID PONE-D-24-21114

Dear Dr. Ventura,

Thank you for granting us the opportunity to do a second review of our manuscript titled "Emotional Skills and Health Assessment in Interventions for Intimate Partner Violence Perpetrators: A Systematic Review of Randomized Controlled Trials", and resubmit it to PLOS ONE.

We have carefully addressed the issues raised by the reviewers, and a detailed point-by-point response is provided on the following pages. Revisions to the manuscript have been marked in the upload file 'Revised Manuscript with Track Changes'. An unmarked version of our revised paper without tracked changes has also been uploaded in a separate file labeled as 'Manuscript'.

If you have any additional questions or further requests, please do not hesitate to contact us. We look forward to your feedback.

Sincerely,

The authors

Journal requirements' and comments of the Editor

Thank you very much for the opportunity to revise our manuscript and for the valuable feedback provided by the reviewers. We greatly appreciate the thoughtful comments and constructive suggestions, which have contributed to improving the quality and clarity of our work.

We have carefully reviewed the manuscript and implemented the recommended revisions. Specifically, we have made changes related to the reference list, the abstract, and the terminology concerning IPV perpetrators. Revisions have also been made to the Introduction, Method, and Results sections to enhance the overall rigor and coherence of the manuscript.

In addition, we have prepared a detailed point-by-point response addressing each of the reviewers' comments. Extended responses to specific concerns—particularly those raised by Reviewer 4—are included in the accompanying response document.

We hope that the revised version of the manuscript meets the expectations of the editorial team and reviewers, and we remain at your disposal for any further clarifications or modifications.

Reviewers’ comments to the authors and responses

Reviewer 4

Comment 1. *The manuscript requires a revision of text for readability and flow, perhaps with assistance from a copy-editor, for the introduction in particular.*

> **Response:** We appreciate your observation regarding the readability and flow of the manuscript, particularly in the Introduction section. To ensure the quality of these revisions, we sought the assistance of a professional academic copy-editor, who provided detailed editorial support. The editor's certificate has been included as part of the manuscript submission.

Comment 2. […] *the main overview of studies is not presented within the study, as recommended by previous reviewers, but rather than as supplementary material.*

> **Response:** Thank you for your comment. The main overview of the studies has been incorporated into the main text. A table titled “Summary of the Main Characteristics of the Final Sample” has been included in the Results section.

Comment 3 (Title). *The title of the paper does not match the aim as described in the abstract (i.e. that the researchers were looking at health assessment also).*

> **Response:** Thank you for this insightful comment. It prompted us to reflect on how we had originally articulated the objectives in the abstract. As the reviewer rightly noted, the abstract did not adequately convey the equal importance of both objectives. This may have given the impression that examining whether programs use health improvement measures as indicators of change and as motivation for intervention adherence was a secondary aim. We have revised the wording to clearly state both objectives of the review. We hope this modification ensures greater consistency with the title and the content developed throughout the manuscript.

Comment 4 (Terminology). *I had comments about interchangeable use of terms for perpetrators also. My recommendation would be to further keep the definition to IPV perpetrator rather than switching terms (i.e. replace ‘aggressor’). I concur with earlier reviewer that ‘batterer’ is archaic and also not inclusive of the broad range of the population included in the review.*

Response: In accordance with the reviewer's comment, we have reviewed the terminology used throughout the text and replaced the relevant terms with *IPV perpetrators*. To improve readability and avoid excessive repetition of the term within the same paragraph, we have also used the word *participants* where appropriate. Additionally, we consulted with the academic copy-editor for guidance on this matter.

Comment 5 (Introduction-A). *The general structure of the introduction is okay, but some more specific explanation of the research gaps in terms of measuring effectiveness of emotional skill building (i.e. a sentence or two summarizing what has been done from reviews, etc., and the rationale for only including RCT's would be useful.*

Response: We appreciate your comment. Both aspects have been revised in the *Introduction*. We have specified and expanded upon the limitations of intervention programs with regard to the incorporation and evaluation of emotional skills. Concerning the use of RCTs, we have sought to strengthen the justification for their selection. In both cases, citations have been included to support the revisions.

Comment 6 (Introduction-B). *The sentence commencing line 101 is problematic: 'In this sense, it would be more productive to ask what content is truly effective and how it works, rather than focusing on a particular approach or type of intervention.' I find hard to comprehend and implies that approach or intervention type are unimportant, whereas they could instead argue that further analysis of content would benefit intervention research. If indeed it is not so productive to discuss, why is there almost a page of text on this in the introduction?*

Response: Thank you for your comment. Your observation is very timely and, indeed, we agree that clarifying this idea and introducing it from the perspective you suggest provides greater coherence with the manuscript's line of argument.

Comment 7 (Introduction-C). *C. Although revised down from the 100+ words in original version, sentence commencing line 64 still 50+ words long.*

Response: In accordance with the reviewer's comment, the sentence has been reduced.

Comment 8 (Introduction-D). *Sentence commencing line 129 lacking supporting references. I suggest checking throughout introduction for this issue.*

Response: Thank you for the comment. Indeed, the statement highlighted by the reviewer requires evidence-based support. To strengthen the argument, we have added two references: Santirso et al. (2020) [30], who discuss motivational strategies that enhance treatment adherence and emphasize the importance of "helping individuals find their own reasons for change"; and Soleymani et al. (2018) [31], who report that motivational interviewing improves motivation or readiness for change, engagement in treatment, and session attendance.

Comment 9 (Methods-A). *Suggest changing 'was not written down' to 'was not published' – it is clearly written down within this manuscript!*

Response: Changed in the text, as suggested by the reviewer.

Comment 10 (Methods-B). *The sentence commencing line 180 could be removed as it really belongs in results.*

Response: The sentence has been removed from the Methods section and incorporated—with adjusted wording—into the Results section.

Comment 11 (Methods-C). *Line 156. States 'd) they reported details of the theoretical approach' – this is the first mention of this – if studies were only included if theoretical underpinning was described, I presume that many useful studies may have been excluded? If this is simply about terminology, then perhaps change this to intervention type or style to clarify. Otherwise, this idea needs to be included in introduction and the reason why theoretical approach was specified as an inclusion criteria clearly spelled out.*

Response: We appreciate your insightful comment. We have replaced the term in the text with "intervention type." Indeed, that is the concept we intended to convey.

Comment 12 (Results-A). *Under 'study selection' the additional study identified via bibliographic review is mentioned both at the beginning and end of this section. One should be removed.*

Response: The duplicated information has been removed.

Comment 13 (Results-B). *In general, there are large tracts of text that could instead be referred to in the table that is currently supplied as a supplementary material. I strongly encourage that the table is included within the manuscript, and that the description of the models, the study duration, the control/reference populations, and the follow-up periods*

could be reduced to 1-2 sentences each, with reference to the table. This would ensure the reader is more easily directed to the more pertinent results (i.e. the main outcomes and trial authors findings and conclusions)

Response: Thank you for your helpful comment. In response, we have integrated a summary table outlining the main characteristics of the studies directly into the text. Furthermore, following the reviewer's suggestion, we revised the Results section to eliminate redundancy, with the aim of enhancing clarity and improving the manuscript's overall readability. Specifically, priority has been given to information that helps address the objectives of our research and will later inform the discussion—namely, details related to the content of the interventions and their outcomes. The remaining information has been briefly summarized, with only selected aspects of interest presented at the end of that section.

Comment 14 (Results-C). *Line 270 can you perhaps provide a different description of master-level therapist? I have no idea what this means – is it a counsellor or psychologist?*

Response: Thank you for your comment. As the information in the Results section was reduced, the concept "*master-level therapist"* has been removed.

Comment 15 (Results-D). *Dialectical behavioral therapy was not presented in the introduction, and probably should as intro makes it looks like CBT and Duluth are the only approaches.*

Response: Thank you very much for your comment. Dialectical Behavior Therapy (DBT) is a form of cognitive-behavioral therapy (CBT) that emphasizes the development of concrete skills, particularly those aimed at learning to manage intense emotions and impulses. DBT was not specifically discussed in the introduction because its core components and foundational principles are derived from CBT, which was addressed in that section. To clarify this point, Table 1 includes this information so that readers can properly situate DBT within the broader framework of CBT.

Comment 16 (Discussion and conclusion). *The discussion reads well and I had nothing to add to the comments and changes already made. The figures and table look fine too (I just think the table belongs in the body of manuscript).*

Response: Thank you very much for your comment. The table has been incorporated in the body of the manuscript.

Reviewer 5

We thank Reviewer 5 for their thorough review of the manuscript and for the valuable feedback provided.
