# Supplementary material for: Emotional skills and health assessment in interventions for intimate partner violence perpetrators: A systematic review of randomized controlled trials
Source: PLoS One. 2025 Jul 9;20(7):e0328034. doi: 10.1371/journal.pone.0328034 (PMC12240372; doi:10.1371/journal.pone.0328034)
Supplement: Attachment — Submitted filename: Response to Reviewers-3.pdf [file pone.0328034.s008.pdf]

Emotional Skills and Health Assessment in Interventions for Intimate Partner Violence Perpetrators: A Systematic Review of Randomized Controlled Trials

Manuscript ID PONE-D-24-21114

Dear Dr. Ventura,

Thank you for granting us the opportunity to do a third review of our manuscript titled "Emotional Skills and Health Assessment in Interventions for Intimate Partner Violence Perpetrators: A Systematic Review of Randomized Controlled Trials", and resubmit it to PLOS ONE.

We have carefully addressed the Journal requirements', and a detailed point-by-point response is provided on the following pages. Revisions to the manuscript have been marked in the upload file 'Revised Manuscript with Track Changes'. An unmarked version of our revised paper without tracked changes has also been uploaded in a separate file labeled as 'Manuscript'.

If you have any additional questions or further requests, please do not hesitate to contact us. We look forward to your feedback.

Sincerely,

The authors

Journal requirements’

Comment 1. *Please review your reference list to ensure that it is complete and correct. If you have cited papers that have been retracted, please include the rationale for doing so in the manuscript text, or remove these references and replace them with relevant current references. Any changes to the reference list should be mentioned in the rebuttal letter that accompanies your revised manuscript. If you need to cite a retracted article, indicate the article’s retracted status in the References list and also include a citation and full reference for the retraction notice.*

Response: Thank you for the recommendation. We have reviewed the reference list and corrected the errors identified in the numbering order of the in-text citations and corresponding references. In this third revision, aside from addressing the aforementioned issue, no citations have been added or removed. Nonetheless, we note that such changes were made during the previous revision of the manuscript (Revision 2).

- The study by Babcock (2004) has an updated version of the meta-analysis; therefore, the citation has been replaced with the more recent one:

  23. Babcock JC, Gallagher MW, Richardson A, Godfrey DA, Reeves VE, D’Souza J. Which battering interventions work? An updated Meta-analytic review of intimate partner violence treatment outcome research. Clin Psychol Rev 2024;111:102437. pmid: 38810357

- The following new citations have been included:

  31. Santirso FA, Gilchrist G, Lila M, Gracia E. Motivational strategies in interventions for intimate partner violence offenders: A systematic review and meta- analysis of randomized controlled trials. Psychosoc Interv 2020;29(3):175-190.

  32. Soleymani S, Britt E, Wallace-Bell M. Motivational interviewing for enhancing engagement in intimate partner violence (IPV) treatment: A review of the literature. Aggress Violent Behav 2018;40:119-127.

  33. Zarling A, Berta M, Miller A. Changes in Psychological Inflexibility and Intimate Partner Violence Among Men in an Acceptance and Commitment Therapy-Based Intervention Program. Behav Sci 2025;15:317. pmid: 40150212

53. Turner W, Morgan K, Hester M, Feder G, Cramer H. Methodological Challenges in Group-based Randomised Controlled Trials for Intimate Partner Violence Perpetrators: A Meta-summary. Psychosoc Interv 2023;32:123–136. pmid:37383642

54. Karakurt G, Koç E, Çetinsaya E. E, Ayluçtarhan Z, Bolen S. Meta-analysis and systematic review for the treatment of perpetrators of intimate partner

Comment 2. *While revising your submission, please upload your figure files to the Preflight Analysis and Conversion Engine (PACE) digital diagnostic tool, https://pacev2.apexcovantage.com/. PACE helps ensure that figures meet PLOS requirements. To use PACE, you must first register as a user. Registration is free. Then, login and navigate to the UPLOAD tab, where you will find detailed instructions on how to use the tool. If you encounter any issues or have any questions when using PACE, please email PLOS at figures@plos.org. Please note that Supporting Information files do not need this step.*

Response: We have proceeded to upload the Figures, as indicated in the comment, to the Preflight Analysis and Conversion Engine (PACE) digital diagnostic tool. A screenshot of the process can be seen below.

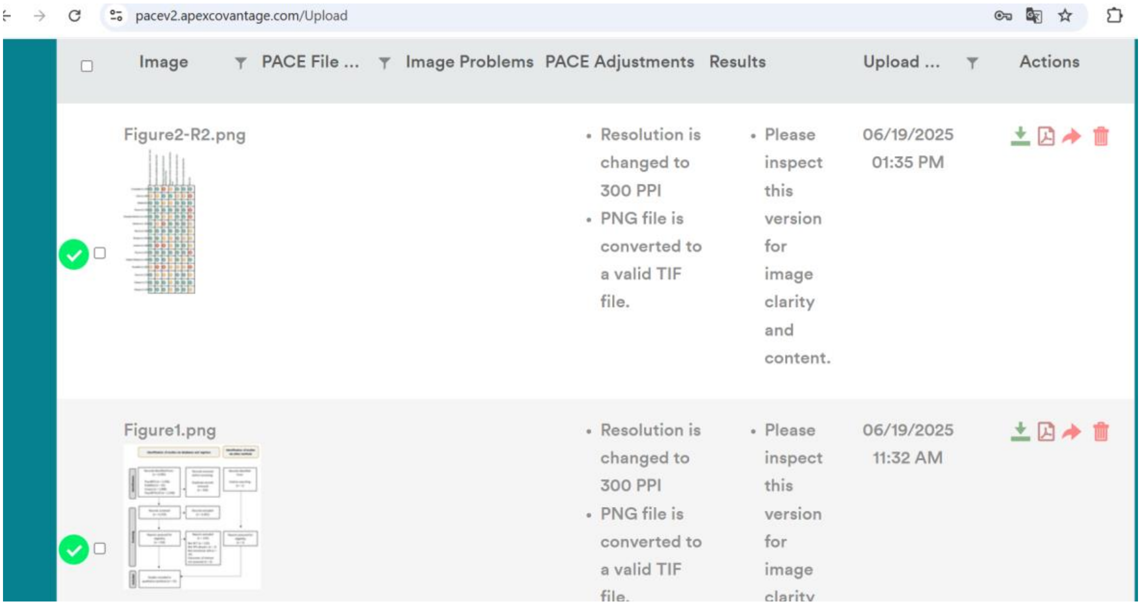

Lorem Ipsum

Lorem ipsum dolor sit amet, consectetur adipiscing elit. Mauris maximus fringilla ligula, in malesuada erat tempor ac. Quisque dapibus posuere turpis, vel aliquam massa vehicula non.

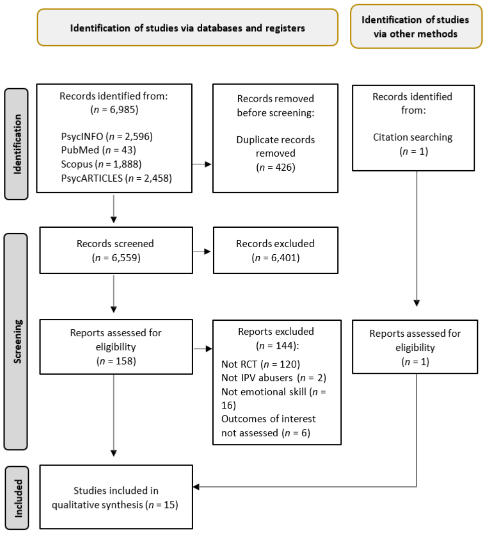

Figure1_202506193246.tif This is a preview of your figure rendered on a simulated PLOS journal page.

Maecenas ac est sit amet odio sollicitudin euismod. In risus odio, convallis a neque ac, varius ultricies arcu. Vestibulum et quam iaculis, ultricies odio et, molestie magna. Suspendisse vehicula purus id turpis eleifend, et convallis dui dignissim. Praesent tempus elit a metus sollicitudin, sed fringilla nulla porttitor. Nullam in tempus massa. Nunc maximus magna massa, nec volutpat risus rhoncus ut. Fusce quis ante sem. Aenean nulla nibh, tempus sit amet rhoncus at, eleifend vel risus. Sed dictum, sem ultrices elementum pharetra, lacus diam volutpat orci, scelerisque semper dui lacus ut enim.

Suspendisse in nunc id lacus commodo consequat. Proin semper aliquam varius. Fusce vitae neque aliquam nisi ultrices sodales vitae ut enim. Vivamus nec dictum ipsum. Sed condimentum ante eu urna tincidunt tincidunt. In ac lacus nec ipsum viverra volutpat posuere vel lacus. Class aptent taciti sociosqu ad litora torquent per conubia nostra, per inceptos himenaeos. Morbi rhoncus ipsum quis lorem hendrerit, at vulputate massa tempus. Ut arcu nisl, gravida vitae risus ultricies, porta venenatis massa. Cras dignissim, enim at faucibus aliquam, sapien nisl eleifend dolor, vel mollis nulla nisi id ipsum. Pellentesque vehicula ultricies risus sit amet faucibus. Praesent sit amet mi ac est faucibus accumsan. Praesent pulvinar sit amet orci auctor feugiat.

Phasellus vitae congue est. Duis rutrum iaculis nunc, sed sollicitudin neque eleifend nec. Pellentesque ac nisi eget tortor imperdiet sagittis ut in orci. Mauris porta convallis euismod. Donec in ultricies urna, nec interdum lectus. Nullam sit amet finibus augue, eget rutrum metus. Nam faucibus, urna ac finibus eleifend, neque nisi lobortis ante, at pharetra purus purus sed urna. Curabitur sit amet dui at enim porta posuere non vehicula ligula. Suspendisse potenti. Vestibulum arcu magna, vulputate a massa ac, molestie tincidunt dui.

Donec id tempus lacus, sed tristique nulla. Nullam rutrum risus ut pharetra porttitor. Nam mattis dolor erat, sed volutpat est mattis sed. Suspendisse eu porta tellus. Cras gravida velit sed maximus fermentum. Fusce vitae metus commodo, sagittis nunc sed, faucibus nunc. Integer iaculis quam mattis, luctus neque in, viverra magna. Nulla rhoncus feugiat orci, quis posuere ligula ornare at. Integer vel sagittis risus. Donec semper metus nec finibus accumsan. Mauris sit amet suscipit ante. Aliquam accumsan, nisl vitae vulputate elementum, turpis nibh varius urna, vel bibendum nulla nunc ac quam. Aenean malesuada egestas maximus. Pellentesque faucibus, odio at tincidunt ullamcorper, eros nisi pellentesque mi, non blandit sapien neque quis lectus.

Lorem Ipsum

Lorem ipsum dolor sit amet, consectetur adipiscing elit. Mauris maximus fringilla ligula, in malesuada erat tempor ac. Quisque dapibus posuere turpis, vel aliquam massa vehicula non.

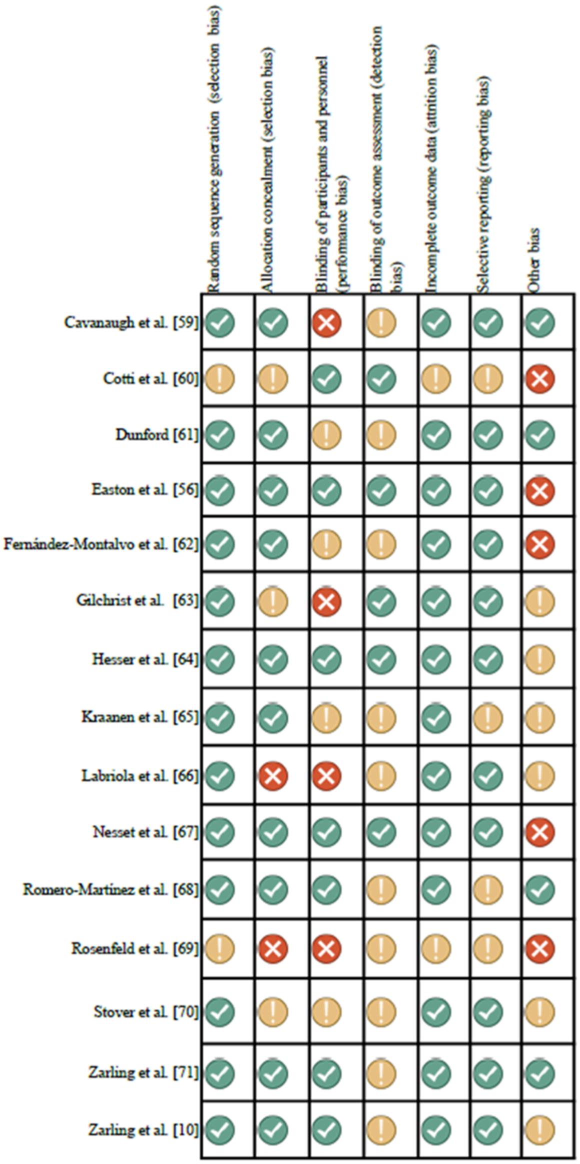

Figure2-R2_202506193525.tif This is a preview of your figure rendered on a simulated PLOS journal page.

Maecenas ac est sit amet odio sollicitudin euismod. In risus odio, convallis a neque ac, varius ultricies arcu. Vestibulum et quam iaculis, ultricies odio et, molestie magna. Suspendisse vehicula purus id turpis eleifend, et convallis dui dignissim. Praesent tempus elit a metus sollicitudin, sed fringilla nulla porttitor. Nullam in tempus massa. Nunc maximus magna massa, nec volutpat risus rhoncus ut. Fusce quis ante sem. Aenean nulla nibh, tempus sit amet rhoncus at, eleifend vel risus. Sed dictum, sem ultrices elementum pharetra, lacus diam volutpat orci, scelerisque semper dui lacus ut enim.

Suspendisse in nunc id lacus commodo consequat. Proin semper aliquam varius. Fusce vitae neque aliquam nisi ultrices sodales vitae ut enim. Vivamus nec dictum ipsum. Sed condimentum ante eu urna tincidunt tincidunt. In ac lacus nec ipsum viverra volutpat posuere vel lacus. Class aptent taciti sociosqu ad litora torquent per conubia nostra, per inceptos himenaeos. Morbi rhoncus ipsum quis lorem hendrerit, at vulputate massa tempus. Ut arcu nisl, gravida vitae risus ultricies, porta venenatis massa. Cras dignissim, enim at faucibus aliquam, sapien nisl eleifend dolor, vel mollis nulla nisi id ipsum. Pellentesque vehicula ultricies risus sit amet faucibus. Praesent sit amet mi ac est faucibus accumsan. Praesent pulvinar sit amet orci auctor feugiat.
