# Supplementary material for: Emotional skills and health assessment in interventions for intimate partner violence perpetrators: A systematic review of randomized controlled trials
Source: PLoS One. 2025 Jul 9;20(7):e0328034. doi: 10.1371/journal.pone.0328034 (PMC12240372; doi:10.1371/journal.pone.0328034)
Supplement: Attachment — Submitted filename: Referee report_2024_09_30.docx [file pone.0328034.s003.docx]

Referee report

This systematic review studies the small literature evaluating batterer intervention programs and other perpetrator-focused DV prevention protocols. The authors limit attention to randomized control trials of programs for perpetrators of IPV, synthesizing evidence from 15 published studies. The authors find that studies of perpetrator programs show stronger results when the curriculum involves a substantial amount of socioemotional and empathy development.

The study is only able to speak to relative effectiveness of BIPs versus actual effectiveness relative to other policy levers. Because it limits attention to RCTs, which are often infeasible in contexts studying IPV/DV, the ultimate sample size is small, and the reader is left with questions about generalizability of findings. As discussed below, I also find that the headline result is unsupported by the data included.

The nascent state of the literature in question (RCTs on IPV perpetrator interventions with and without emotional skill development) leads me to question whether this is an appropriate moment for a systematic review. The methods used seem over-powered by the size of the sample, whereas a more informal literature review could easily ascertain differences between these fifteen studies without spending unnecessary length on describing Cochrane methods, etc.

Major comments

- The choice to limit attention to RCTs is problematic. Excluding 120 out of 158 full-text articles because they used methods other than RCT feels shortsighted. RCT is often an impractical or even unethical choice of method when studying violence, as randomizing individuals who perpetrate and/or experience violence into treatment has potential to create new harms. Some studies in the sample have treatment group sizes of 28, which absolutely precludes any chance of sufficient statistical power to detect significant effects. Yet, the authors claim that such studies are more ‘rigorous’ than all other studies using other methods (pg 22).
- The authors make the following claim as a headline finding: “Overall, the results of this systematic review indicated that more than half (53.3%) of interventions for perpetrators of GBV that included the acquisition of emotional skills were significantly more effective in reducing recidivism and improving variables associated with this type of violence than interventions that did not focus on developing these competencies.” As a reader, I interpret this to mean that of those interventions that include emotional skills, 53% had larger effect sizes found than the studies of interventions that did not include emotional skills. This is a coin toss—at the sample size included in the paper, there is no way to claim a statistically significant difference between those emotional intervention studies that do and do not find larger effects. Equally half of studies on emotional interventions found larger and smaller impacts than the other studies. If my interpretation is untrue, then the paper needs to be substantially revised such that it is clear to the reader what the authors are claiming. If my interpretation is correct, then claiming that emotional interventions are associated with better outcomes is patently unsupported by the data.
- In multiple places the results presented in the discussion are inconsistent with the results presented in the findings section or with such significant caveats as to undermine the initial results.
- Recidivism data—it is unclear how these data came about in the fifteen studies chosen. Only five studies use outcomes on recidivism from official channels, which is subject to considerable bias. The other use self-report or do not have information on recidivism. This should be addressed explicitly as anti-social behaviors are subjected to reporting bias, especially if the BIP is court mandated.
- Which studies included voluntary enrollment versus court-mandated programming? How would the effects generalize to populations where BIP is required as an outcome of legal proceedings, which may directly impact participants’ willingness to meaningfully engage?
- The methods limit the inclusion of research articles to those studying IPV specifically, but the earlier portions of the text refer to “battering” or broader studies on violence. If IPV is the focus of this paper, specific language should be used and a definition should be provided that make clear the inclusion/exclusion criteria.
- Pg 6 “e) they provided recidivism rates or described the outcomes for **any factor associated with intimate partner violence** (IPV)” What does this mean? It’s unclear what the outcome variable inclusion criteria were.
- Pg 10 “seven trials (46.7%) included voluntary participants who were not involved in a relationship characterized by a severe form of violence or abuse, therefore, there were no convictions for IPV in those trials” What does this mean? Why were these participants recruited?

Minor comments

- Current preference is to avoid using language like “batterer” because it limits attention to physical violence. (An exception to this would be so-called ‘batterer intervention programming’ as this is a term of practice rather than a choice by the authors.)
- “Regarding random sequence generation, two trials (13.3%) did not describe a random component in the sequence generation.” Explain what this means and its significance.
- “their intervention group showed an IPV success rate” what does this mean?
- Acronyms are used without first defining them.
- Provide more information on what the Duluth Model consists of.
- The draft is difficult to follow in places with a number of parentheticals within the same sentence, for example.
